# Distressed Breathing in a Cow1Read before the annual meeting of the Pennsylvania State Veterinary Medical Association, March, 1898.

**Published:** 1898-04

**Authors:** J. F. Butterfield

**Affiliations:** Montrose, Pa.


### DISTRESSED BREATHING IN A COW.[1]

By J. F. Butterfield, V.S.,
MONTROSE, PA.

In April, 1891, I introduced a tracheotomy-tube into the trachea of a two-year-old registered Jersey heifer belonging to Mr. M. W.

[1] Read before the annual meeting of the Pennsylvania State Veterinary Medical Association, March, 1898.

Palmer, of Kingley, Susquehanna County, Pa., to relieve the acute distressed breathing caused from internal and external swelling in a case of laryngitis or pharyngitis. She wore the tube from Sunday until Friday, when it was taken out, the swelling having subsided and breathing normal. The wound healed in due time.

About two years ago this same patient showed signs of distressed or labored breathing. It continued, and gradually grew worse until last July, when it was continuous. Could hear her several rods away; in fact, it was very distressing to hear her. Owner said I must remedy this in some way, so that it would be no trouble to him. Upon examination I found the two tracheal rings that were cut to introduce the treacheotomy-tube had flattened, nearly closing the trachea. To remedy this lesion I made a piece of No. 12 hard or spring silver wire into a speculum. I cast the cow in the usual manner, opened the two flattened tracheal rings, as in the first operation six years before, introduced the wire, and with a piece of soft silver wire fastened the upper end of the speculum to one of the tracheal rings that had not been cut, closed the wound with antiseptic dressings, etc.

On the cow regaining her feet she went out and commenced feeding as though nothing had happened. Not a cough or any symptoms of distressing breathing. Her owner, Mr. Palmer, writes me, March 3, 1898: "The cow is doing well, and in as good health and breathes as well as any in the barn; she dropped a nice heifer-calf in September, since the operation, which I am raising."

